# 基于Ti_3_C_2_T*_x_*/聚酰亚胺复合材料的分散固相萃取-液相色谱法测定尿液中儿茶酚胺类神经递质

**DOI:** 10.3724/SP.J.1123.2022.09004

**Published:** 2023-07-08

**Authors:** Yuanqing ZHAO, Kai HU, Cheng YANG, Pengzhao HAN, Lixin LI, Xiaobing LIU, Zhenqiang ZHANG, Shusheng ZHANG

**Affiliations:** 1.河南中医药大学中医药科学院,河南郑州450046; 1. Academy of Chinese Medical Sciences, Henan University of Chinese Medicine, Zhengzhou 450046, China; 2.郑州大学现代分析与基因测序中心,河南郑州450001; 2. Center of Advanced Analysis and Gene Sequencing, Zhengzhou University, Zhengzhou 450001, China

**Keywords:** 液相色谱, 分散固相萃取, 二维碳化钛, 聚酰亚胺, 儿茶酚胺类神经递质, liquid chromatography (LC), dispersive solid-phase extraction (DSPE), two dimensional titanium carbide (Ti_3_C_2_T*_x_*), polyimide, catecholamines (CAs)

## Abstract

研究通过一种快速、简便的方法制备了聚酰亚胺功能化的二维碳化钛复合材料Ti_3_C_2_T*_x_*,并用作分散固相萃取吸附剂,结合液相色谱-荧光分析方法对尿液样品中痕量儿茶酚胺类神经递质(CAs)进行分离和分析。利用多种手段对Ti_3_C_2_T*_x_*/聚酰亚胺的形貌、性质等进行了表征,并详细考察了萃取参数对Ti_3_C_2_T*_x_*/聚酰亚胺萃取儿茶酚胺类神经递质的萃取性能的影响,结果表明,该复合材料可以通过静电、*π-π*和氢键作用有效富集目标化合物。最佳萃取条件如下:吸附剂用量为20 mg、样品pH为8.0、吸附时间和脱附时间分别为10 min和15 min、解吸溶剂为醋酸-乙腈-水(5∶47.5∶47.5, v/v/v)。将Ti_3_C_2_T*_x_*/聚酰亚胺用作分散固相萃取吸附剂与HPLC-FLD联用,建立了一种尿液中CAs的灵敏检测方法,实现了4种CAs物质的定量分析。在最优的条件下,该方法中去甲肾上腺素、肾上腺素、多巴胺和异丙肾上腺素的线性范围为1~250 ng/mL,相关系数(*r*^2^)均大于0.99,检出限LOD(*S/N*=3)在0.20~0.32 ng/mL之间,定量限LOQ(*S/N*=10)在0.7~1.0 ng/mL之间,日内精密度相对标准偏差(RSD)在0.7%~1.09%之间,日间精密度相对标准偏差(RSD)在1.73%~4.24%之间,在实际样品中的加标回收率在82.50%~96.85%之间,精密度RSD的范围在2.47%~9.96%之间。基于Ti_3_C_2_T*_x_*/聚酰亚胺的分散固相萃取-液相色谱法具有萃取速度快、灵敏度高等特点,可以成功用于尿液中CAs的检测分析。

## Abstract (en)

Neurotransmitters (NTs) are basic signaling chemicals used for communication between cells. The most well-known catecholamines (CAs) are epinephrine, norepinephrine, and dopamine. CAs are an important class of monoamine NTs that contain catechins and amine groups. The accurate determination of CAs in biological samples can provide essential information on potential pathogenic mechanisms. However, biological samples generally contain only trace levels of CAs. Therefore, sample pretreatment is necessary to separate and enrich CAs before instrument analysis. Dispersive solid-phase extraction (DSPE) technology combines the principles of liquid-liquid extraction and solid-phase extraction and is a useful method for purifying and enriching the target analytes in complex matrices. This method has the advantages of low solvent consumption, environmental safety, and high sensitivity and efficiency. In addition, the adsorbents used in DSPE do not need to be packed into a column and can simply be completely dispersed in the sample solution; this excellent feature greatly improves the extraction efficiency and simplifies the extraction process. Therefore, the development of new DSPE materials with high efficiency and adsorption capacity using simple preparation procedures has received wide attention from the research community. Carbon nitrides (MXenes) are a class of two-dimensional layered materials that possess good hydrophilicity, a large number of functional groups (-O, -OH, and -F), large layer spacing, different elemental compositions, excellent biocompatibility, and environmental friendliness. However, these materials have a small specific surface area and poor adsorption selectivity, which limits their applications in SPE. The separation selectivity of MXenes can be significantly improved by functional modification. Polyimide (PI) is a crosslinking product that is mainly formed by the condensation polymerization of binary anhydride and diamine. It has a unique crosslinked network structure, as well as a large number of carboxyl groups, and shows excellent characteristics. Therefore, the synthesis of new PI-functionalized Ti_3_C_2_T*_x_* (Ti_3_C_2_T*_x_*/PI) composites by growing a PI layer on the surface of two-dimensional MXene nanosheets in situ may not only overcome the adsorptive limitations of MXenes but also effectively improve their specific surface area and porous structure, thereby enhancing their mass transfer capacity, adsorption capacity, and selectivity.

In this study, a Ti_3_C_2_T*_x_*/PI nanocomposite was fabricated and successfully applied as a DSPE sorbent to enrich and concentrate trace CAs in urine samples. The prepared nanocomposite was examined using various characterization methods, including scanning electron microscopy, Fourier transform-infrared spectroscopy, X-ray diffraction, and zeta potential analysis. The effects of the extraction parameters on the extraction efficiency of Ti_3_C_2_T*_x_*/PI were also investigated in detail. The adsorption performance of Ti_3_C_2_T*_x_*/PI can be described by pseudo-second-order kinetics and the Freundlich isotherm model. The adsorption process appeared to occur on the outer surface, as well as surface voids, of the nanocomposite. The adsorption mechanism of Ti_3_C_2_T*_x_*/PI indicated a chemical adsorption process based on multiple electrostatic, *π-π*, and hydrogen-bonding interactions. The optimal adsorption conditions included an adsorbent dosage of 20 mg, sample pH of 8, adsorption and elution times of 10 and 15 min, respectively, and eluent composed of acetic acid-acetonitrile-water (5∶47.5∶47.5, v/v/v). A sensitive method for detecting CAs in urine was subsequently developed by coupling Ti_3_C_2_T*_x_*/PI as a DSPE sorbent with HPLC-FLD analysis. The CAs were separated on an Agilent ZORBAX ODS analytical column (250 mm×4.6 mm, 5 μm). Methanol and an aqueous solution of 20 mmol/L acetic acid were used as the mobile phases for isocratic elution. Under optimal conditions, the proposed DSPE-HPLC-FLD method exhibited good linearity in the range of 1-250 ng/mL with correlation coefficients >0.99. The limits of detection (LODs) and limits of quantification (LOQs) were calculated based on signal-to-noise ratios of 3 and 10 and found to be in the range of 0.20-0.32 and 0.7-1.0 ng/mL, respectively. The recoveries of the method were in the range of 82.50%-96.85% with RSDs≤9.96%. Finally, the proposed method was successfully applied to the quantification of CAs in urine samples from smokers and nonsmokers, thereby indicating its applicability for determining trace CAs.

神经递质(neurotransmitters, NTs)是细胞间用来交流的基本化学物质之一。它们在机体应对压力的反应、运动协调、精神运动控制、稳态功能以及神经元间的通讯方面发挥着核心作用^[1,2]^。根据结构的不同,这些化合物可分为单胺类神经递质和氨基酸类神经递质等^[3]^。儿茶酚胺类神经递质(catecholamines, CAs)是一类重要的单胺类神经递质,主要包括肾上腺素(adrenaline, E)、去甲肾上腺素(noradrenaline, NE)和多巴胺(dopamine, DA),在神经系统方面发挥着重要的作用,并表现出强大的生理功能,可作为多种神经和心血管疾病的诊断、治疗和预防的生物标志物^[4]^。目前研究已经证实,NE和E在糖尿病、心脏病和焦虑症的研究中充当生物标志物,而DA的缺乏已被证实与帕金森病和精神分裂症有关^[5,6]^。因此,对生物样品中CAs物质进行精准检测,可以为研究人员提供有关潜在致病机制的重要信息,反映治疗效果,从而为临床合理用药乃至新的药物设计和新治疗方法提供科学依据^[7]^。

现有的检测生物样本中CAs的方法主要是仪器分析方法,包括电化学传感器、高效液相色谱(HPLC)^[8]^、高效毛细管电泳(HPCE)^[9]^和液相色谱-质谱联用(LC-MS)^[7]^等。尽管目前用于检测生物基质中CAs的分析方法取得了很大的进展,但生物样品中CAs的含量一般较低,而且基质比较复杂,因此需要进行样品前处理,从而对其进行分离富集^[10,11]^。固相萃取(SPE)技术是目前使用最广泛的样品前处理技术之一,具有成本低、使用方便、灵敏高效、环境友好等优点^[12,13]^,适用于多种生物样品中被测组分的富集。它主要利用固体吸附介质对目标物进行萃取吸附,使目标物与基质及干扰物分离,从而达到分离和富集目标物的目的^[14]^。目前,文献中已经报道了多种用于生物样品中CAs分离和富集的固相萃取材料,包括分子印迹聚合物^[13]^、石墨烯量子点改性的铜网电极^[1]^、亲水亲油平衡(HLB)材料涂层的薄膜等^[7]^。然而,SPE存在扩散和传质速度有限、处理时间长、吸附柱堵塞等问题。在分散固相萃取(DSPE)中,吸附剂不装在柱中,而是完全分散到样品中,可以大大提高萃取效率,简化萃取过程。同时DSPE技术结合液液萃取和固相萃取的原理,利用吸附剂吸附杂质,溶剂用量少,具备环保、灵敏度高、高效快速等优点,因此开发制备简便、高效、吸附性强的新型分散固相萃取材料受到了相关从业者的广泛关注。

碳氮化物(MXene)是一种二维(2D)类石墨烯材料,它的化学式为M*_n_*_+1_X*_n_*T*_x_*(n=1、2或3),其中M表示过渡金属(例如Ti、Mo、V、Zr和Cr等), X表示碳、硼或氮,T*_x_*代表表面上的末端官能团,例如-OH、-O和-F等^[15]^。MXene因其独特的化学性质和独特的性能而受到极大的关注,这源于它们具有二维层状形态、亲水性以及大量的官能团(-O、-OH和-F),同时还有大的层间距、不同的元素组成、出色的生物相容性和环境友好性^[16]^。研究人员基于MXene的性质,将其应用于生物、环境、气体储存等领域^[17⇓⇓-20]^。MXene本身的缺点,如比表面积小、吸附选择性差等,限制了其在固相萃取领域的广泛应用。通过制备MXene复合材料,对其进行功能化修饰,可以提高其分离选择性,提高富集效率。聚酰亚胺类(PI)是主链含酰亚胺结构的一类人工高分子材料的总称,常见的有联苯型、均苯型、酮酐型、醚酐型。均苯型聚酰亚胺主要是由二元酸酐与二元胺经过缩合聚合反应制成的一种交联物,由于其主链上含有的芳环结构,同时具备特殊的交联网状结构以及大量的羧基基团而表现出优异的特性^[21]^。PI上大量的芳环结构可以与CAs物质产生强的*π-π*作用,同时丰富的羧基可以与CAs上的羟基有一定的氢键作用,羧基能提供一个负电荷的状态与带氨基的CAs产生静电吸附,提高MXene对CAs的分离选择性。目前,基于MXene复合材料用于固相萃取的研究鲜有报道,将聚酰亚胺修饰到二维碳化钛Ti_3_C_2_T*_x_*纳米片表面,可以有效提高Ti_3_C_2_T*_x_*表面的功能基团,有望进一步提高其对生物样本中CAs类物质的分离选择性。

本研究设计合成了一种Ti_3_C_2_T*_x_*/聚酰亚胺复合材料(Ti_3_C_2_T*_x_*/PI),并以其作为分散固相萃取材料,结合HPLC-FLD,建立了尿液中儿茶酚胺类神经递质的分离分析方法,实现了尿液中儿茶酚胺类神经递质的分离分析,拓展了MXene功能化材料在分离分析领域的应用。

## 1 实验部分

### 1.1 仪器与试剂

Agilent 1290超高效液相色谱(德国Agilent公司),配备G1321B型荧光检测器;MIRA LMS场发射扫描电镜(捷克Tescan公司); Nicolet710傅里叶变换红外光谱仪(美国尼高力公司); Empyrean X射线衍射仪(荷兰Panalytical B.V.公司); 90Plus PALS高灵敏度Zeta电位及粒度分析仪(美国Brookhaven公司); H2-16K台式高速离心机(湖南可成仪器设备有限公司); PHS-3C型pH计(上海仪电科学仪器股份有限公司); KQ5200DE型超声机(昆山市超声仪器有限公司)。

NE、E、DA、异丙肾上腺素(isoproterenol, ISO)、均苯四甲酸酐(PDMA)、对苯二胺、3-氨丙基三乙氧基硅烷(APTES)、*N*-甲基吡咯烷酮(NMP)、氢氧化钠(NaOH)均购自阿拉丁试剂有限公司(上海);乙醇(EtOH)、氨水(NH_3_·H_2_O)、乙酸(HAc)、甲酸(FA)购自麦克林试剂有限公司(上海);盐酸(HCl)购自Sigma公司(上海);甲醇(MeOH)、乙腈(ACN)购自Fisher Scientific公司(美国);在实验中使用的超纯水(≥18.2 MΩ·cm)均通过Milli-Q系统过滤;末端带有羟基的Ti_3_C_2_T*_x_*购自费曼纳米科技(郑州)。

标准溶液配制:准确称取NE、E、DA、ISO标准品各10 mg,分别用MeOH定容至10 mL,配成质量浓度为1 mg/mL的储备液,所有溶液均置于4 ℃的冰箱中备用。

实际样品:非吸烟者和吸烟者尿样由河南中医药大学(年龄>21岁,各3份)提供(已经过伦理委员会批准,编号为:2022HL-399),分析前将尿液置于冰箱-20 ℃保存。

### 1.2 色谱条件

采用Agilent ZORBAX ODS色谱柱(250 mm×4.6 mm, 5 μm)进行色谱分离。柱温为25 ℃,进样量为20 μL,流动相由MeOH和20 mmol/L HAc水溶液(12∶88, v/v)组成,以1.0 mL/min的流速等度洗脱。荧光检测器的激发波长和发射波长分别设置为280 nm和330 nm。所有样品用0.22 μm微孔滤膜过滤后进样。

### 1.3 Ti_3_C_2_T*_x_*/PI材料的制备

Ti_3_C_2_T*_x_*-NH_2_的制备:准确称取500 mg Ti_3_C_2_T*_x_*分散在80 mL乙醇中,然后加入5.0 mL H_2_O和7.5 mL NH_3_·H_2_O,并在室温下搅拌24 h。随后,向混合物中加入2.0 mL APTES,继续60 ℃反应搅拌24 h。使用H_2_O和乙醇交替清洗3次,并在60 ℃真空下过夜干燥。

Ti_3_C_2_T*_x_*/PI的制备:准确称取300 mg Ti_3_C_2_T*_x_*-NH_2_和281.4 mg PDMA分散于15 mL NMP中,超声分散均匀后,磁力搅拌1 h,然后继续加入281.4 mg PDMA和139.5 mg对苯二胺,磁力搅拌1 h后转移至高压反应釜中,200 ℃高温反应12 h,然后用H_2_O和乙醇交替清洗复合材料3次,然后在60 ℃真空下过夜干燥。具体合成过程及样品萃取过程如图1所示。

**图1:**
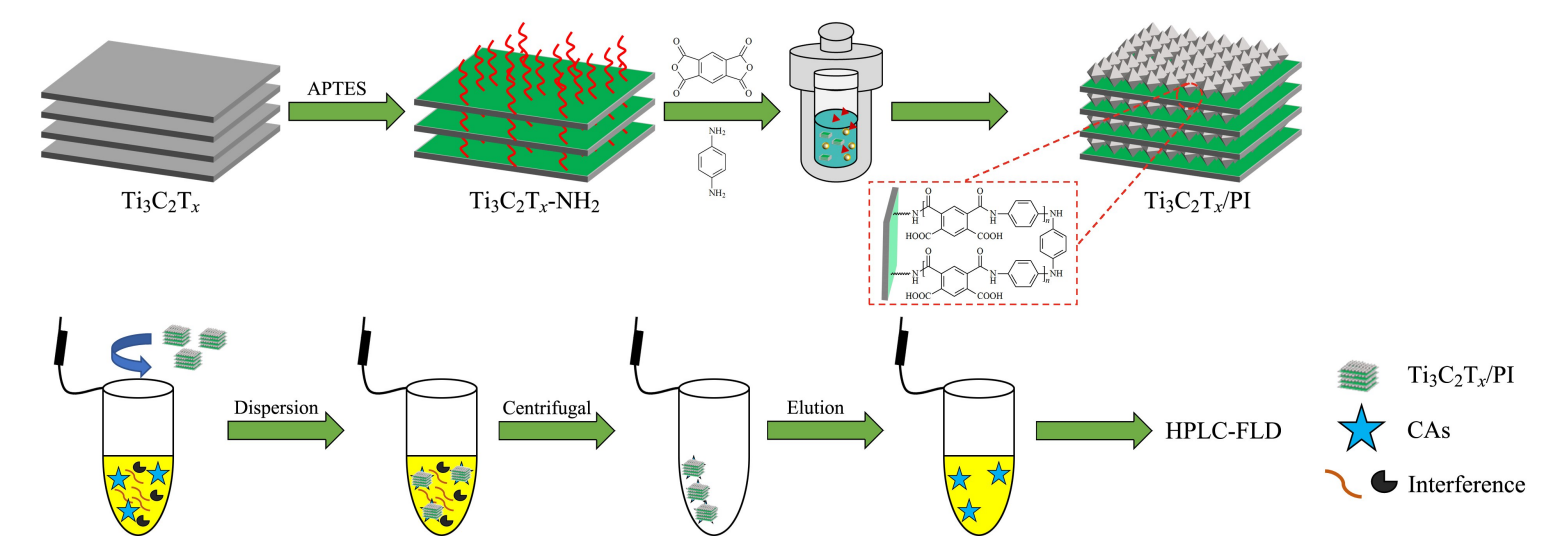
Ti_3_C_2_T*_x_*/PI复合材料的制备过程及样品萃取过程

### 1.4 实际样品预处理

于室温下解冻尿样,将100 μL ACN加入5.0 mL的尿样中,在4 ℃下离心15 min(12000 r/min)以沉淀蛋白质。之后收集上清液,取尿液1.0 mL,用水稀释至2.5 mL,制成实际样品溶液。

### 1.5 萃取过程

取2.5 mL实际样品溶液,用0.1 mol/L NaOH溶液调节pH为8.0,称取20.0 mg Ti_3_C_2_T*_x_*/PI复合材料,加入溶液中,超声吸附10 min后离心1 min(12000 r/min),弃去上清液,加入2.5 mL醋酸-乙腈-水溶液(5∶47.5∶47.5, v/v/v)超声15 min后离心1 min(12000 r/min),收集解吸液,重复解吸3次后合并解吸液。用旋转蒸发仪40 ℃减压浓缩至近干,用0.5 mL醋酸-乙腈-水溶液(5∶47.5∶47.5, v/v/v)复溶,经0.22 μm滤膜过滤后再使用HPLC-FLD进行分析检测。

## 2 结果与讨论

### 2.1 Ti_3_C_2_T*_x_*/PI的表征

采用扫描电镜对Ti_3_C_2_T*_x_*/PI的形貌和颗粒尺寸进行表征,如图2a和2b所示,在放大的倍数下,可以观察到层状的Ti_3_C_2_T*_x_*表面覆盖许多丝状的交联物,而且比较均匀。同时能量色散光谱图见图2c~f,可知N元素均匀地分布在复合材料中,说明交联物PI均匀地分布在Ti_3_C_2_T*_x_*的表面,表明复合材料的成功制备。

**图2:**
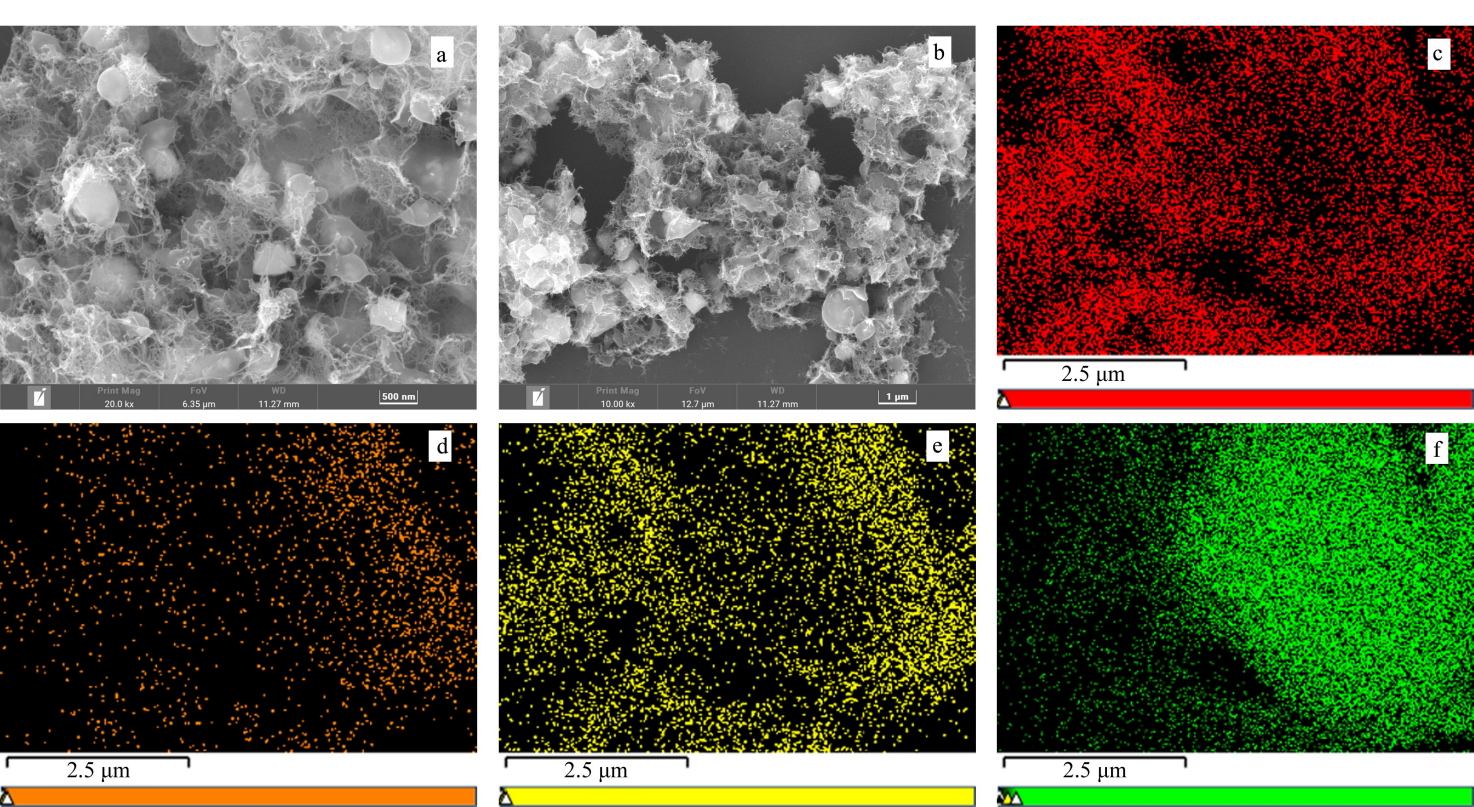
Ti_3_C_2_T*_x_*/PI的(a, b)SEM图和(c, d, e, f)能量色散光谱图

如图3a所示,Ti_3_C_2_T*_x_*的IR光谱在3427 cm^-1^处有吸收,归属于-OH伸缩振动;在570、1046 cm^-1^处的峰对应于Ti-O和C-O振动;Ti_3_C_2_T*_x_*-NH_2_在3500、3400 cm^-1^波长处归属于N-H振动,同时新出现的特征峰Si-O和Si-O-Ti的伸缩振动分别为1042、874 cm^-1^,说明成功接枝硅烷偶联剂。Ti_3_C_2_T*_x_*/PI在红外光谱中可以看到明显的苯环吸收峰,分别对应1520、1450、802、718 cm^-1^波长处;而且羧基的信号峰也较强,3482和1706 cm^-1^波长处的吸收峰归属于-OH和C=O的振动吸收,表明PI交联物已成功地包覆在Ti_3_C_2_T*_x_*表面。

**图3:**
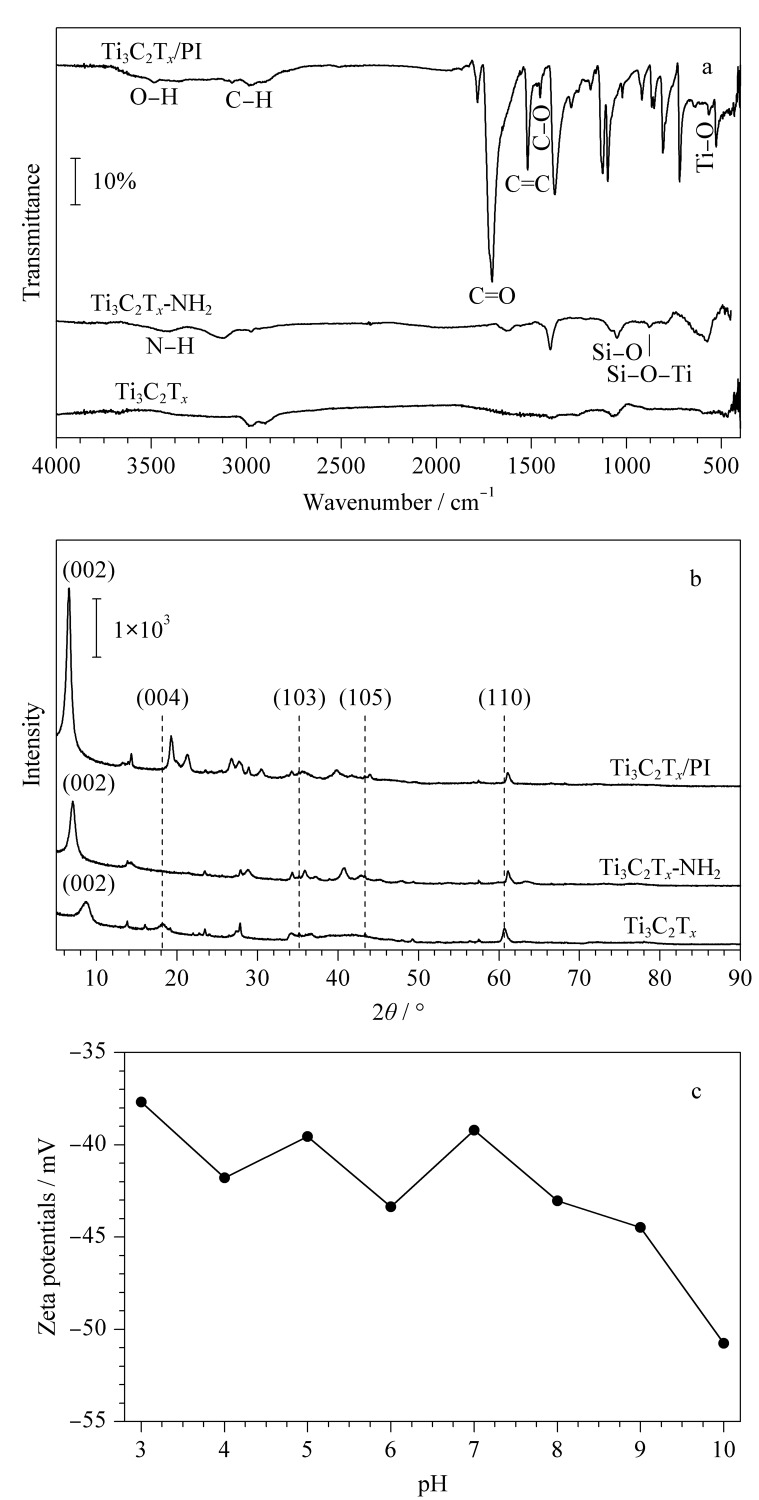
Ti_3_C_2_T*_x_*、Ti_3_C_2_T*_x_*-NH_2_和Ti_3_C_2_T*_x_*/PI的(a)红外 光谱图、(b)X射线衍射图谱和(c)Ti_3_C_2_T*_x_*/PI的Zeta电位图

Ti_3_C_2_T*_x_*、Ti_3_C_2_T*_x_*-NH_2_和Ti_3_C_2_T*_x_*/PI的X射线衍射图如图3b所示。Ti_3_C_2_T*_x_*衍射图在8.73°、18.45°、36.03°、43.72°和60.72°处显示出典型峰,对应于(002)、(004)、(103)、(105)和(110)晶格平面。Ti_3_C_2_T*_x_*-NH_2_的(002)衍射峰从8.73°偏移到7.12°,同时衍射峰的强度增加,说明修饰后纳米片层距离发生改变。Ti_3_C_2_T*_x_*/PI的(002)衍射峰继续发生偏移至6.45°,同时19.21°、21.42°等处增加新的衍射峰,这表明Ti_3_C_2_T*_x_*-NH_2_高温反应之后晶型发生了一定程度改变,结合扫描电镜和红外光谱结果,证明已经形成了Ti_3_C_2_T*_x_*/PI复合材料。

从文献^[22]^中得知,对于原始Ti_3_C_2_T*_x_*纳米片,其Zeta电位在4~10时随着pH的增加而降低,在pH 6左右达到零电荷点;在pH>6.0时Ti_3_C_2_T*_x_*的负电荷表面可以归因于Ti_3_C_2_T*_x_*表面上各种基团的影响,例如-F和Ti-O-基团。相比之下,如图3c所示,Ti_3_C_2_T*_x_*/PI的Zeta电位在pH 3~10范围内电位均为负值,数值远远低于Ti_3_C_2_T*_x_*,这是因为PI交联物包覆在Ti_3_C_2_T*_x_*表面,其含有丰富的羧基基团可以在pH 3~10的范围内去质子化而带负电荷,负的Zeta电位将有利于通过静电吸引吸附各种阳离子物质。

### 2.2 萃取条件的优化

为了获得最佳萃取性能,本研究对可能影响Ti_3_C_2_T*_x_*/PI萃取效率的一系列参数,如吸附时间、样品pH、吸附剂用量、解吸溶剂和解吸时间进行了优化。优化过程中使用质量浓度为500 ng/mL的4种CAs混合标准溶液,并通过目标物回收率评估吸附和解吸性能。

#### 2.2.1 吸附时间

吸附时间是影响萃取性能的重要因素,足够的吸附时间可以使样品中的目标物与吸附剂充分作用,从而提高萃取效率。实验中考察了不同吸附时间(0~45 min)对萃取性能的影响。如图4a,当吸附时间由0增加到10 min时萃取效率不断上升,10 min时基本达到吸附平衡,接着延长吸附时间,萃取效率基本保持不变,因此后续实验的萃取时间选为10 min。

**图4:**
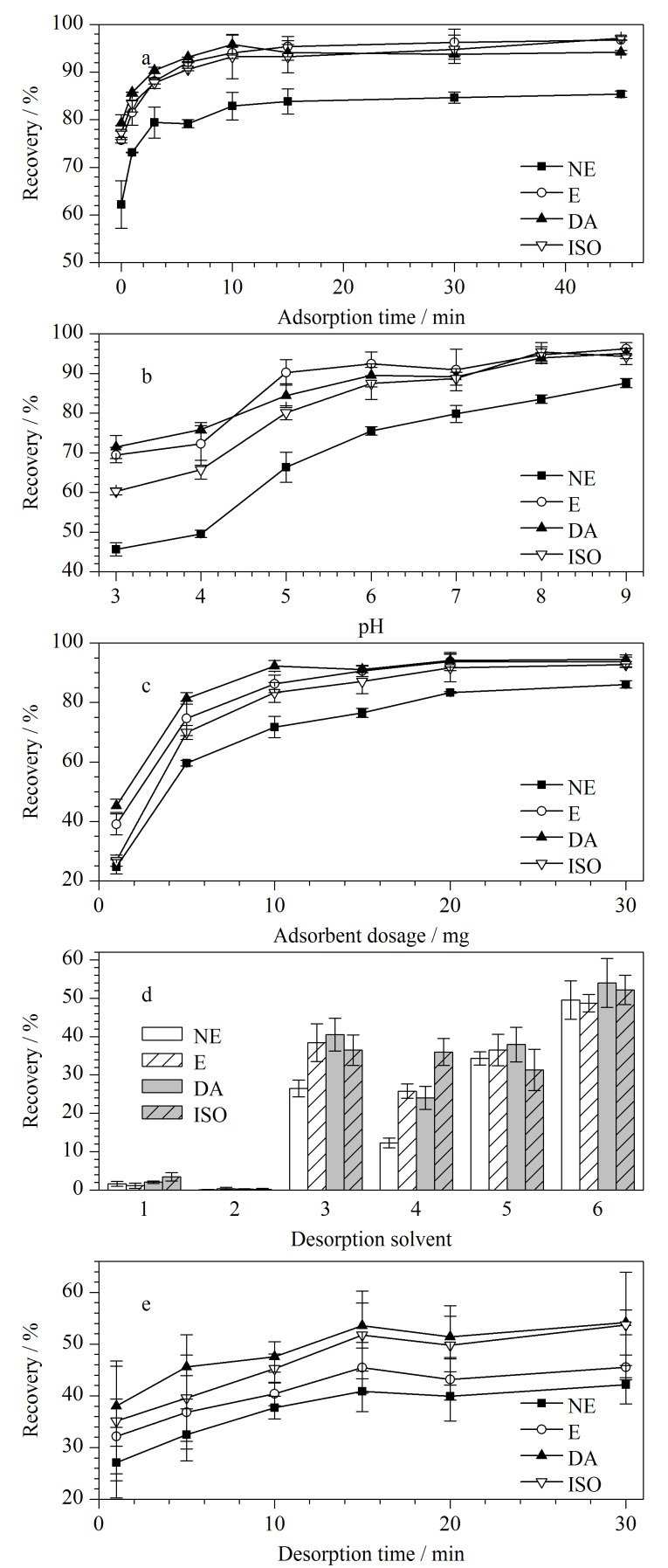
(a)吸附时间、(b)样品pH、(c)吸附剂用量、(d)解吸溶剂、(e)解吸时间对目标物回收率的影响(*n*=3)

#### 2.2.2 样品pH

溶液的pH影响着Ti_3_C_2_T*_x_*/PI复合材料表面的电荷和目标物的性质,从而影响吸附性能。如图4b,在低pH时,吸附剂上的羧基未发生解离,复合物与目标物之间静电作用较弱,主要靠吸附剂上的*π-π*作用吸附CAs;随着pH的升高,羧基开始发生解离,复合物与目标物之间静电作用增强,萃取效率提高。此外,目标物带有的氨基、羟基等基团与复合物上的杂原子之间还会存在氢键作用,也有利于吸附效率的提高。考虑到CAs在高pH环境下易氧化分解,所以选择pH 8.0为最优pH进行后续实验。

#### 2.2.3 吸附剂用量

考察了吸附剂用量(1~30 mg)对萃取性能的影响。由图4c可知,随着吸附剂用量的不断增加,萃取效率随着吸附剂用量的增加而增强,E、DA、ISO 3种物质在20 mg时达到最佳效果,继续增加吸附剂用量对萃取效果无明显影响,因此选择20 mg为最佳吸附剂用量并用于后续实验。

#### 2.2.4 解吸条件

Ti_3_C_2_T*_x_*/PI复合材料对CAs的吸附作用主要是靠静电吸附、*π-π*作用和氢键作用,在解吸过程中为了破坏相关吸附作用力,考虑使用有醋酸的有机溶剂来解吸目标物。如图4d所示,选择甲醇、乙腈、5%醋酸甲醇溶液、5%醋酸乙腈溶液、醋酸-甲醇-水(5∶47.5∶47.5, v/v/v)和醋酸-乙腈-水(5∶47.5∶47.5, v/v/v)为解吸溶剂,比较发现醋酸-乙腈-水(5∶47.5∶47.5, v/v/v)的解吸效果最好。

然后考察解吸时间对解吸效率的影响。如图4e所示,解吸时间为1~15 min时,解吸效率逐渐增加到40.85%~53.6%之间,后续继续增加解吸时间,解吸效率没有明显升高,所以选择解吸时间为15 min。为了将目标物充分洗脱下来,使用上述解吸溶液解吸3次后解吸效率在90%以上,故将解吸次数定为3次,最优的解吸条件即为以醋酸-乙腈-水(5∶47.5∶47.5, v/v/v)解吸3次,每次15 min。

### 2.3 吸附性能和吸附机理考察

#### 2.3.1 吸附动力学

进行吸附动力学研究可以提供吸附机理和吸附特性的相关信息。吸附行为通常使用准一级和准二级两个动力学方程来描述。准一级动力学方程为ln (*q*_e_-*q_t_*)=ln *q*_e_-*k*_1_*t*,准二级动力学方程为*t/q_t_=*1*/*(*k*_2_ ${q_{e}}^{2}$ )+*t/q*_e_。其中*q*_e_和*q_t_*是平衡时间和时间*t*(min)时的吸附容量(mg/g), *k*_1_是准一级吸附的速率常数(min^-1^), *k*_2_是准二级吸附速率常数(g/(mg·min))。

基于准一级和准二级动力学的CAs在Ti_3_C_2_T*_x_*/PI上的吸附研究结果如图5所示,使用准二级吸附动力学方程对相关数据进行拟合,相关系数均在0.99以上,远高于一级动力学拟合的相关系数,说明准二级动力学方程更适合用于描述CAs在Ti_3_C_2_T*_x_*/PI的吸附动力学行为。由图5b也可以看出,准二级动力学模型拟合的数据具有良好线性关系,表明吸附可能发生在外表面和表面的空隙上,属于化学吸附,主要靠表面的电荷作用进行吸附。

**图5:**
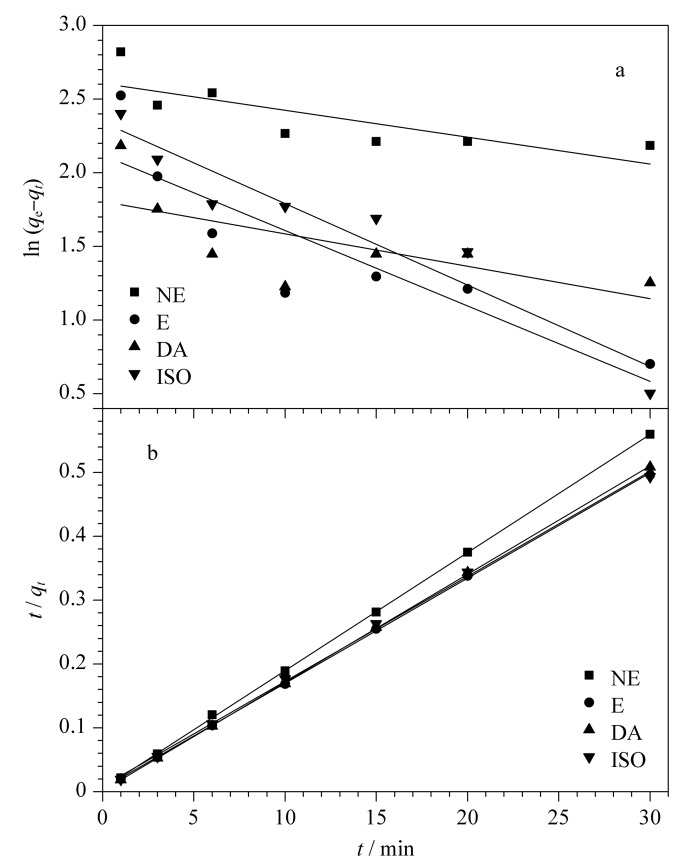
Ti_3_C_2_T*_x_*/PI对CAs的吸附动力学

#### 2.3.2 吸附等温线

吸附等温线通常用于描述平衡吸附机制。Langmuir和Freundlich吸附等温线模型是使用最广泛的两种模型,其分别基于单层吸附和多层吸附。Langmuir模型方程为*q*_e_=*C*_e_*q*_0_*b*/(*C*_e_*b*+1), Freundlich模型为*q*_e_=*K*_f_ ${C_{e}}^{1/n}$ 。其中*C*_e_(mg/L)是CAs的平衡浓度,*b*(L/mg)是Langmuir常数,*q*_0_(mg/g)为最大吸附容量。*q*_e_(mg/g)为平衡吸附时CAs的量,*K*_f_为Freundlich常数。

图6是两种等温线模型的拟合结果,Freundlich模型拟合的结果相关系数均大于0.97,明显优于Langmuir等温线模型(相关系数在0.89~0.92之间)。因此,Freundlich模型的相关系数较高,表明复合材料表面是不均一的,在吸附过程中,CAs在Ti_3_C_2_T*_x_*/PI表面形成多分子层并对其进行吸附。本研究考察了CAs在1~20 μg/mL时的吸附等温线,研究了4种CAs在该质量浓度下对Ti_3_C_2_T*_x_*/PI吸附容量的影响,Ti_3_C_2_T*_x_*/PI复合材料对4种CAs的吸附容量随着溶液初始浓度的增加而升高,在初始质量浓度为15 μg/mL时基本达到饱和,Ti_3_C_2_T*_x_*/PI对4种CAs的最大吸附容量在2.13~2.36 mg/g之间。

**图6:**
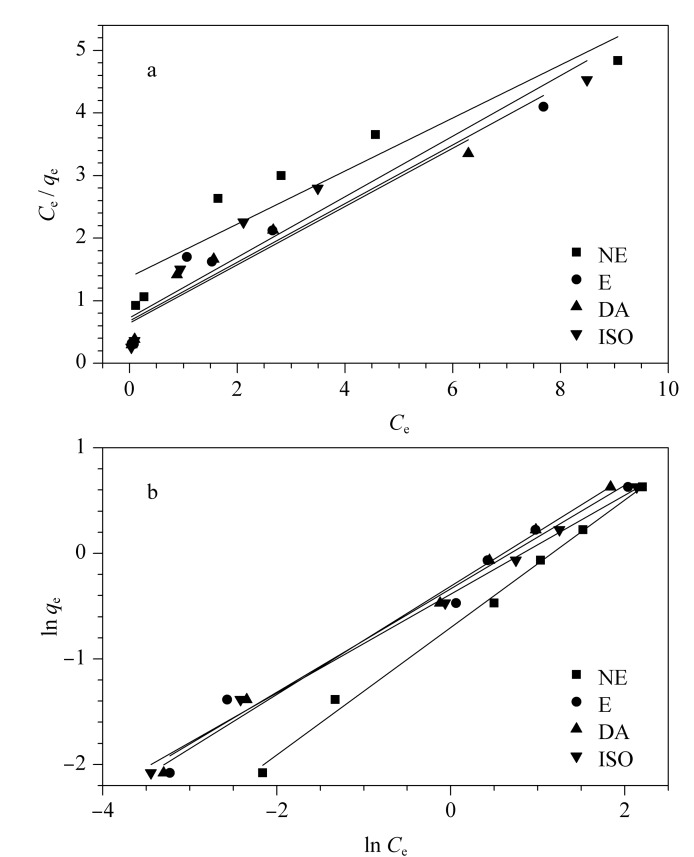
Ti_3_C_2_T*_x_*/PI对CAs的吸附平衡等温线

### 2.4 方法学考察

在最佳萃取条件下,向空白尿液样品中加入不同浓度的目标分析物,经萃取和HPLC-FLD分析后建立标准曲线。4种CAs的线性范围在1~250 ng/mL之间,所有标准曲线具有良好的线性相关性(相关系数*r*^2^均大于0.99), LOD和LOQ分别在0.20~0.32 ng/mL和0.7~1.0 ng/mL之间。

1天内连续制备6组同一质量浓度样品进样分析,再连续3天制备同一质量浓度样品进样分析,分别得到日内和日间精密度。日内的精密度(*n*=6)在0.57%~1.09%之间,日间的精密度(*n*=3)在1.73%~4.24%之间,说明该分析方法的适用性较好。

4种CAs的线性范围、检出限(LOD, *S/N*=3)、定量限(LOQ, *S/N*=10)及日内、日间精密度结果见表1。

**表1:** 4种CAs的线性方程、相关系数、线性范围、检出限、定量限和相对标准偏差

Analyte	Linear equation	*r*^2^	Linear range/ (ng/mL)	LOD/ (ng/mL)	LOQ/ (ng/mL)	RSDs/%	
						Intra-day (*n*=6)	Inter-day (*n*=3)
NE	*y*=5.68×10^-2^*x*-1.96×10^-2^	0.9982	1.0-250	0.32	1.0	1.09	4.24
E	*y*=5.14×10^-2^*x*-3.48×10^-2^	0.9962	1.0-250	0.30	1.0	0.57	1.73
DA	*y*=5.36×10^-2^*x*+3.72×10^-2^	0.9977	1.0-250	0.20	0.7	0.80	2.64
ISO	*y*=5.75×10^-2^*x*-2.04×10^-2^	0.9931	1.0-250	0.30	1.0	0.59	2.73

*y*: peak area; *x*: mass concentration, ng/mL.

### 2.5 实际样品检测及回收率考察

为验证所建方法的分离检测效果,将其应用于吸烟者和非吸烟者尿液中CAs的检测,结果如表2所示。吸烟者样品中NE、E和DA的含量分别为9.05~13.26、4.65~10.22和11.32~21.58 ng/mL,非吸烟者样品中NE、E和DA的含量分别为1.65~2.00、1.95~2.91、4.98~7.40 ng/mL,未检测到外源性的ISO,根据数据可以看出吸烟者尿液中CAs的含量相对较多。通过在非吸烟者的尿样中加入3个浓度水平(25、100和250 ng/mL)的CAs,考察了方法的回收率和精密度。从表3数据观察可知,加标后4种CAs的相对回收率在82.50%~96.85%之间,日内回收率RSD为2.47%~9.96%。

**表2:** 吸烟者和非吸烟者尿液样品中CAs的含量

Analyte	Smoker				Non-smoker		
	Sample 1	Sample 2	Sample 3		Sample 1	Sample 2	Sample 3
NE	9.05	9.22	13.26		2.00	1.47	1.65
E	10.22	4.65	9.18		1.95	2.91	2.65
DA	21.58	15.8	11.32		6.27	7.40	4.98
ISO	ND	ND	ND		ND	ND	ND

ND: not detected.

**表3:** 4种CAs在尿液中3个水平下的加标回收率和精密度(*n*=3)

Analyte	25 ng/mL			100 ng/mL			250 ng/mL	
	Recovery/%	RSD/%		Recovery/%	RSD/%		Recovery/%	RSD/%
NE	93.31	9.23		82.50	2.47		93.25	2.99
E	96.25	5.53		83.57	3.16		92.67	5.65
DA	87.38	8.07		94.86	4.16		96.85	5.07
ISO	89.15	9.96		92.44	6.04		83.64	2.63

### 2.6 与其他方法的比较

表4总结了所提出的方法与最近发表的用于检测CAs方法的比较。与其他文献报道的方法相比,本研究建立的方法萃取时间仅需要10 min,具有较短的萃取时间。将本研究中的LOD与之前报告的LOD进行了比较。可以看出,与大多数报道的方法相比,本方法显示出相对较低的LOD,这表明本研究中的方法足够灵敏。同时本研究方法的回收率和精密度较好,表明所开发的方法具有快速、灵敏、可靠的优点,具备实际应用的潜力。

**表4:** 该方法与参考文献中其他方法的比较

Detection system	Materials	Extraction/ min	Linear range/ (ng/mL)	LODs/ (ng/mL)	Recoveries/ %	RSDs/ %	Ref.
HPLC-FLD	magGO@POSS-BA	2	10-500	0.54-2.28	81.3-101.7	6.9-9.7	[23]
HPLC-FLD	Fe_3_O_4_@SiO_2_/IDA-Cu	30	0.7-50	0.20-0.33	86.2-109.4	3.8-10.0	[24]
HPLC-UV	BA-RAM	30	5-100	-	87-114	7.6-14.4	[25]
LC-MS/MS	ZrO_2_	10	1-200	0.035-0.050	91.0-109.5	2.8-9.4	[26]
HPLC-FLD	Fe_3_O_4_@COF@2-FPBA	60	2-200	0.31-0.54	86.3-114.9	2.34-10.5	[27]
HPLC-FLD	Fe_3_O_4_@Ti_3_C_2_T*_x_*-BA	2	1-500	0.03-0.16	88.14-112.3	3.03-11.7	[28]
HPLC-FLD	Ti_3_C_2_T*_x_*/PI	10	1-250	0.20-0.32	82.50-96.85	2.47-9.96	this work

magGO: magnetized graphene oxide; POSS: polyhedral oligomeric silsesquioxanes; BA: boric acid; IDA: iminodiacetic acid; RAM: restricted-access material; COF: covalent organic framework; 2-FPBA: 2-formylphenylboronic acid; PI: polyimide

## 3 结论

本研究以Ti_3_C_2_T*_x_*/PI作为固相萃取材料,通过分散固相萃取结合HPLC-FLD分析将其应用于尿液中4种CAs的分离检测。结果表明,Ti_3_C_2_T*_x_*/PI对4种CAs具有良好的吸附性能,建立的分析方法成功用于吸烟者和非吸烟者尿液中CAs的检测分析。所建立的方法具有操作简便、萃取时间短、灵敏度高等优点,可以满足检测的需求。本方法为复杂生物基质中痕量CAs常规监测分析提供了新的分析方法。
